# Towards a sustainable, comprehensive and community-accepted nomenclature and naming standard of antibody and T cell receptor germline genes and alleles

**DOI:** 10.3389/fimmu.2025.1689673

**Published:** 2025-11-24

**Authors:** Ayelet Peres, Mats Ohlin

**Affiliations:** 1 Department of Pathology, Yale University, New Haven, CT, United States; 2 Department of Immunotechnology and SciLifeLab, Lund University, Lund, Sweden

**Keywords:** adaptive immune receptor repertoire (AIRR), germline gene, gene nomenclature, reference sets, nomenclature

Technological advances have brought us to a point where we can now observe, interpret, and understand both the capabilities and limitations of the adaptive immune system—particularly its role in defending against external threats and internal challenges such as oncogenic transformation and hostile environments. These developments also enable us to examine how pathological conditions may arise from erroneous actions of the adaptive immune system itself.

Central to these processes is the generation of B cell receptors (BCRs) and antibodies, and T cell receptors (TCRs) by B and T cells, respectively. Together, these molecules form the Adaptive Immune Receptor Repertoire (AIRR), which enables the immune system to recognize and respond to a vast array of antigens. The immense diversity of these receptors, driven by gene diversity, gene rearrangements and, in the case of antibodies, affinity maturation through somatic hypermutation and selection, allows the adaptive immune system to respond to any encountered antigens, provided appropriate co-stimulatory signals are present.

Due to their importance in health and disease, extensive studies of adaptive immunity are carried out, studies that generate vast datasets. Proper interpretation of this data relies on standardized computational tools, as well as a consistent language for analysis and visualization. Numerous tools have been developed and are in widespread use. Recognizing the need for standardization, efforts to catalogue and define the genes involved in generating BCR/antibody and TCR-encoding sequences began more than three decades ago engaging both the international ImMunoGeneTics information system (IMGT) (1) and others (2). These standardized frameworks have greatly facilitated data analysis, interpretation, and communication within the research community. Developments in sequencing technology, in particular long-read sequencing and associated bioinformatics tools now enables valid reconstruction even of complex genomic loci such as those encoding AIRRs (3–7). However, as our understanding of the antibody and TCR loci has deepened—and as research has expanded to include previously understudied human populations and additional species—the challenges of developing complete, valid reference sets of germline genes participating in the generation of BCRs/antibodies and TCRs, while maintaining consistent nomenclature and naming standards, have become obvious (8). Some of these include:

- Multiple Numbering and Naming Schemes: Various systems for definition of the most diverse, commonly antigen-binding parts, the complementarity determining regions (CDRs), of BCRs, antibodies and TCRs, and gene and sequence numbering have been developed [e.g. (9–15)] but also been volatile with respect to their standard definitions. Several of these definitions are still in concurrent use. Multiple systems for gene naming have been implemented over the years. Although naming authority ought to reside with the International Union of Immunological Societies (IUIS) and its Nomenclature Committee, this seems not to be universally accepted. This creates confusion and hinders the consistent interpretation of results. A single naming body ought to be universally accepted.
- Position-Based Gene Naming: Gene names have traditionally been based on genomic position, aiding interpretation. However, growing evidence shows that genomic structures are far more complex than initially believed. Duplicated, inserted, and deleted genes and inverted segments (5, 16–18), absent from early maps, now challenge this approach. Moreover, AIRR loci in many species are significantly more diverse than in humans (19). Experimentally important model systems, like different mouse strains, differ substantially in their germline gene repertoire (20, 21) and germline loci of individuals of rhesus and cynomolgus macaques are very different (22, 23). Such complexity further complicates position-based naming—especially when diversity of the genomic loci structure is incompletely known. A cautious position-based practise of gene naming must be applied if we do not fully comprehend the diversity of these loci. It is even conceivable that the practice of a purely position-based naming scheme must be avoided for some or even most species as it might imply relations and proximity that does not exist.
- Evolving Nomenclature: Changes in gene names and numbering over time have not always been clearly traceable causing challenges in terms of understanding of analysis outcomes. In some cases, identical names have been reassigned to different sequences, introducing ambiguity. Such practices must be avoided; once assigned, a name should never be reused for a different sequence of a given species.
- Unreliable Sequence Naming: Sequence names have sometimes been assigned based on presumed gene location, without solid genomic support. For example, the human allele originally named IGHV4-59*08 recently had to be renamed as an allele of germline gene IGHV4-61, as name assignment that initially was based on conventional sequencing data was challenged by additional analysis (24) and subsequently on an extended genomic analysis.
- Reference sets are in some cases not open-source: Costly licensing agreements may prevent use of appropriate reference sets in commercial products aimed for analysis of AIRR sequence data. Although it is recognised that there is a need to retrieve costs associated to the generation and management of such reference sets, implementation of licencing fees that prevents the universal use of such sets for valid analysis negatively impacts the quality and reproducibility of AIRR research.

To address these issues, we urge all stakeholders in the AIRR research community—including researchers, tool developers, database curators, journal editors, and standard-setting organizations—to agree on and adhere to a common set of principles. These may include:

- A universally recognized nomenclature and naming authority, most likely the T-cell Receptor and Immunoglobulin (TR-IG) Nomenclature Sub-Committee of the IUIS Nomenclature Committee, should govern naming decision. Names approved by this body should be used; unapproved nomenclature should be clearly flagged in any publication or database. The sub-committee’s TR-IG Nomenclature Review Committee has recently released criteria for validating and naming new immunoglobulin and TCR gene/allele sequences (https://wp-iuis.s3.eu-west-1.amazonaws.com/app/uploads/2024/10/04120932/Data.Review.and_.Naming.Dec2023.pdf) as well as an overview of the challenges and likely best approaches to address them (25).
- Universal adoption of a standard sequence numbering system—such as that developed by IMGT (14)—for representing AIRR sequence data. Databases containing AIRR data, including antibody and TCR protein structures, should convert existing entries to conform with this standard, if necessary.
- Only well-documented genes and alleles should be recognised. These should be full-length sequences, primarily those supported by long-read genomic data of sufficient coverage. Discovery by traditional Sanger sequencing, by computational inference of germline allele sequences from transcriptome data (26–28), and by high-throughput gene amplicon sequencing (29), may likely largely be replaced by modern long-read sequencing technologies, while inference may still aid to determine expression levels of inferred alleles. Independently of the methodology used to identify novel genes and alleles, the supporting data should be made fully available to the research community to judge the relevance of the discovery. Inferred sequences from short-read assemblies must not be added to reference sets due to inherent inaccuracies of such analytical processes (30, 31).
- Positional naming of germline genes should only be applied when the structure and diversity of the relevant immunoglobulin or TCR locus in the species under investigation is well understood. Position-based naming must not be applied until a sufficiently representative number of individuals have been genomically assessed to capture population diversity. In many species, diversity may be too extensive for reliable position-based naming to be implemented.
- Germline alleles without defined genomic locations should receive permanent and unique names (32), that do not associate the sequences to specific genes. A diversity of such naming systems have been used in the past but we propose the use of a single unified permanent unique naming structure (32). Importantly, these labels will be retained as easily accessible metadata even when in-depth understanding of the locus in question allows us to assign a permanent, gene-associated name.
- Reference sets of germline alleles and related data used across downstream applications should be made freely available under open-source licenses to ensure their broad usability in tools and pipelines.
- All reports of studies of AIRR data must include version numbers not only of tools but also of reference sets used for the analysis. Such versioned reference sets must be stored indefinitely in publicly available repositories.

We urge all stakeholders in AIRR research to engage in debate, for instance through the processes established within the AIRR Community. This Community within the Antibody Society (https://www.antibodysociety.org/) is a grass-root science community that organises and coordinates researchers and stakeholders engaged in studies of immunoglobulin and TCR repertoires (33). Such open debate will enable the IUIS Nomenclature Committee and the research community too jointly establish and implement collectively agreed-upon principles for reference set management as well as gene naming and nomenclature principles, such as those defined by the TR-IG Nomenclature Sub-Committee. The role of IUIS, the home of the Committee, as a core meeting-point for the scientific community is well established. It engages multiple national and regional societies and associated members, and it is a recognised partner of the World Health Organization and a member of the International Science Council. Its Nomenclature Committee and its subcommittees openly engage the immunology community to define universal and consistent nomenclatures, promoting community-wide acceptance of common principles. Their continued engagement in this process will greatly benefit the research community and our reliance on the quality of reference sequences, and their continued relevance for future data analysis and interpretation processes.
